# Beneficial effects of a polysaccharide-based grinding aid on magnetite flotation: a green approach

**DOI:** 10.1038/s41598-022-10304-x

**Published:** 2022-04-20

**Authors:** Vitalis Chipakwe, Tommy Karlkvist, Jan Rosenkranz, Saeed Chehreh Chelgani

**Affiliations:** Minerals and Metallurgical Engineering, Department of Civil, Environmental and Natural Resources Engineering, Luleå University of Technology, 971 87 Luleå, Sweden

**Keywords:** Materials science, Engineering, Chemical engineering

## Abstract

Grinding is the most energy-intensive step in mineral beneficiation processes. The use of grinding aids (GAs) could be an innovative solution to reduce the high energy consumption associated with size reduction. Surprisingly, little is known about the effects of GAs on downstream mineral beneficiation processes, such as flotation separation. The use of ecofriendly GAs such as polysaccharide-based materials would help multiply the reduction of environmental issues in mineral processing plants. As a practical approach, this work explored the effects of a novel polysaccharide-based grinding aid (PGA) on magnetite's grinding and its reverse flotation. Batch grinding tests indicated that PGA improved grinding performance by reducing energy consumption, narrowing particle size distribution of products, and increasing their surface area compared to grinding without PGA. Flotation tests on pure samples illustrated that PGA has beneficial effects on magnetite depression (with negligible effect on quartz floatability) through reverse flotation separation. Flotation of the artificial mixture ground sample in the presence of PGA confirmed the benefits, giving a maximum Fe recovery and grade of 84.4 and 62.5%, respectively. In the absence of starch (depressant), PGA resulted in a separation efficiency of 56.1% compared to 43.7% without PGA. The PGA adsorption mechanism was mainly via physical interaction based on UV–vis spectra, zeta potential tests, Fourier transform infrared spectroscopy (FT-IR), and stability analyses. In general, the feasibility of using PGA, a natural green polymer, was beneficial for both grinding and reverse flotation separation performance.

## Introduction

Size reduction units (crushing and grinding) in cement and mineral processing plants consume up to 4% of the global electrical energy produced yearly^1^. Grinding, especially in a ball mill as the most popular grinding machine, is a fairly random process, and only 1–2% of the input energy serves to generate the required product sizes^2^. In the cement industry, the use of grinding aids (GAs) has been examined as a promising alternative to address these issues^3,4^. Chemical additives or GAs would be considered as any substance (less than 0.25 wt.%) added to the mill to reduce energy consumption^5–7^. GAs have been mostly examined in the cement industry and are still not widely practiced in mineral beneficiation plants. Based on the cement industry grinding process outcomes, GAs can improve grindability, reduce energy consumption, and increase specific surface area^8–12^. However, the grinding in cement plants is carried out in the last stage of production, and the reduction of the size is the initial step of mineral processing. Thus, the main concerns in the mineral processing plants include the high cost of GAs, potential contamination of the grinding products (purported negative effects on the downstream process), and environmental issues.

The design and selection of GAs are almost exclusively based on their grinding performance. Within the cement industry, many chemicals have been used as GAs. They range from pure chemicals such as triethanolamine (TEA) to more recently high-charge polymers^6,7,9,13^. Polymers are the most commercially existing GAs. They are mainly based on ethylene glycol, propylene glycol, triisopropanol amine (TIPA), triethanolamine (TEA), and tetraethylenepentamine (TEPA)^6,7,14^. Some of these GAs, such as TEPA (amine-based), are nonbiodegradable and raise environmental concerns^15^. Waste streams containing alkanolamines can increase the concentration of ammonia, nitrite, and nitrate, which could infiltrate the subsoils and water sources^15^.

To address current environmental issues, a few investigations have been conducted on using ecofriendly benign materials as GAs. These studies have reported that natural polymers are advantageous because of their low cost, abundance, and nontoxicity. On the other hand, some investigations have explored the utilization of waste streams from other industries such as waste cooking oil, glycerine, lignin, and cane molasses as GAs^16,17^. This has also been motivated by the high cost of triethanolamine-based GAs and the concepts of ‘circular economy’, which are emerging in the production of raw materials to reduce waste generation and reuse of ‘waste’ from other processes. Zhang et al.^17^ demonstrated that a mixture of lignin, cane molasses, and waste glycerine could be used as GAs in cement production. Polysaccharide-based chemistries are a promising alternative to less toxic and cheaper reagent development options^18,19^. They are also organic polymers that are already used as depressants in flotation separation^20–23^.

Since low environmental impact practices are in high demand within the mineral processing value chain^18,24^, the best scenario would be the development of chemicals that improve grinding performance and ensure they do not have adverse impacts on downstream processes. Some studies have focused on the mineral industry with further discussion on downstream effects^25,26^; however, they were not in-depth. Understanding and controlling any GA-separation reagent interactions is critical to ensure that the required downstream process efficiency and integrity of the whole value chain are maintained. Such an understanding would be essential, particularly for flotation separation, where the separation could be efficient in the specific particle size range (mainly −100 + 25 µm)^27,28^.

Previous studies demonstrated the benefits of polysaccharide-based GA (PGA) as a green chemical additive in improving mineral grinding performance^10^ and material rheology^5^. However, the high cost and purported effects on downstream processes as a result of the potential synergistic interaction of the GAs and flotation reagents limit their applications. Moreover, their potential effects on the downstream beneficiation processes such as mineral flotation separation have not been addressed. This study aims to enhance the theoretical insights for using PGA on grinding magnetite and its possible effects on magnetite-quartz flotation separation as a strategic approach. While the new PGA—Zalta™ VM1122 is commercially available, this current work focuses on investigating the interaction of Zalta™ VM1122 with magnetite and flotation reagents through adsorption tests, stability measurements, Fourier transform infrared (FTIR) studies, and zeta potential measurements. Reverse flotation experiments on artificially mixed ore (magnetite + quartz) are presented. Flotation outcomes were used to evaluate the effect of PGA on the process recovery and grade and compare with conventional flotation (without GAs) as a benchmark.

## Materials and methods

### Chemicals

**Figure 1:**
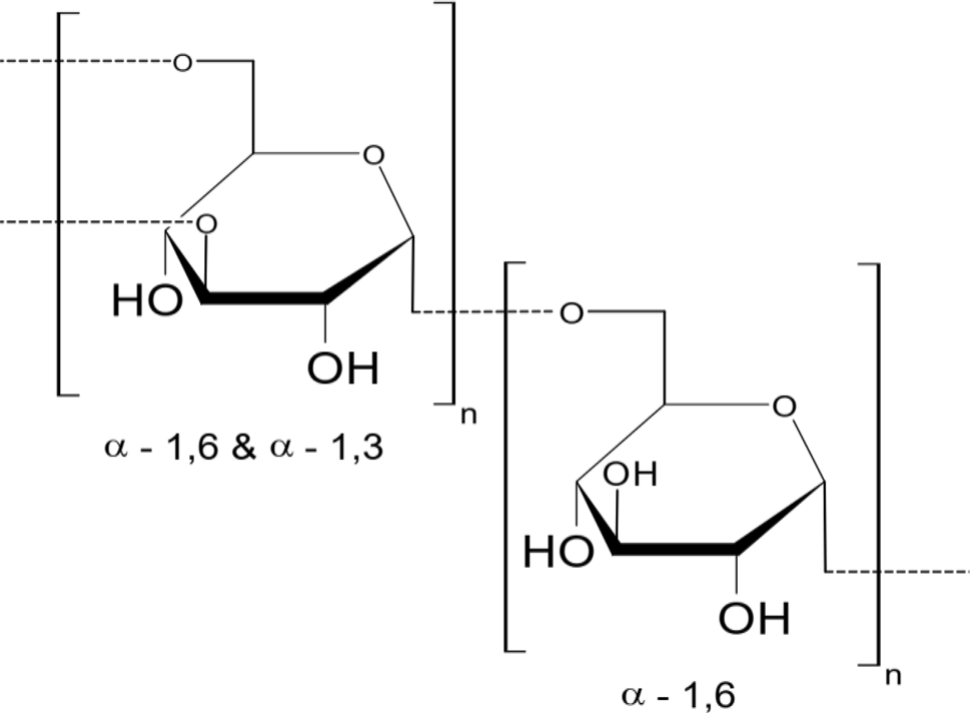
Typical chemical structure of a dextran.

A polysaccharide-based grinding aid (PGA) with the trade name Zalta™ VM1122 was provided by Solenis (Sweden). PGA is a medium-molecular weight polysaccharide that mainly comprises dextran (Fig. 1). For all experiments, the PGA stock solution was freshly prepared daily to avoid any degradation. For the flotation tests, a collector, depressant, and pH modifiers were used (Table 1). Deionized water was used in all experiments unless otherwise noted.

**Table 1:** Materials used for various experiments.

Chemical	Description	Classification	Charge	Source
Zalta™ VM1122 (PGA)	Grinding aid	Polysaccharide-based	Non-ionic	Solenis
Lilaflot 822M	Collector	Ether-amine	Cationic	Nouryon
Starch	Depressant	Corn-starch	–	Merck
Sodium hydroxide	pH modifier	Alkaline	Neutral	Merck
Hydrochloric acid	pH modifier	Acidic	Neutral	Merck

### Minerals

**Figure 2:**
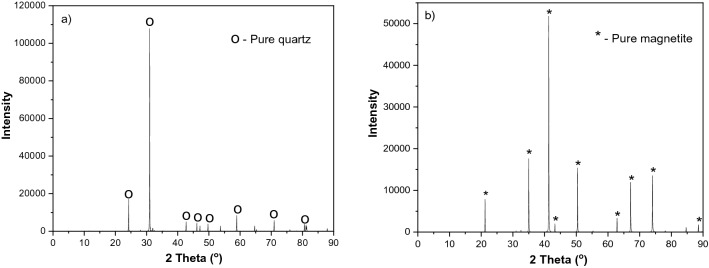
XRD pattern for the examined samples (**a**) pure quartz; (**b**) pure magnetite.

For the experiments, a pure quartz sample (−2 mm) was obtained from VWR, Sweden (Fig. 2a). Magnetite ore from a mine in Malmberget, north of Sweden (Fig. 2b), was received from LKAB (Luossavaara Kiirunavaara Aktiebolag). Semi-quantitative X-ray diffraction (XRD) analyses shows > 99% SiO_2_ for quartz and > 96% Fe_3_O_4_ for magnetite. Magnetite was crushed to −2.8 mm for grinding experiments using a laboratory jaw crusher to obtain mill feed. The pure minerals (magnetite and quartz) were ground using a laboratory ball mill to give −106 µm particle size for flotation and surface analyses. The resulting −106 + 38 µm fraction was used as flotation feed, while −38 µm material was further ground using a mortar and pestle to obtain −5 µm material for surface analyses.

### Grinding

For the grinding experiments, a laboratory-scale ball mill (CAPCO, UK) of 115 mm internal diameter was operated at 91% critical speed, with steel grinding media (graded charge: top size 36 mm). In the control tests, no additives (referred to as ‘reference’) were used, and for the other experiments, PGA was combined with ore at three different concentrations (0.03, 0.05, and 0.1 wt.%). Mill conditions were kept constant for all runs and replicates. The particle size distribution (PSD) was determined using a combination of dry and wet sieve analysis using standard sieves and a RO-TAP® sieve shaker (model RX-29-10, W.S. Tyler, Mentor, OH, USA) from which the P_80_ was determined. Energy consumption was characterized using the work index according to Bond’s Eqiuation^29^. Surface area was measured using the Brunner Emmet Teller (BET) technique by the Micromeritics Flowsorb II 2300 instrument, which characterizes the surface area of the particles using nitrogen gas. Furthermore, the surface area was used to calculate the surface roughness (${R}_{S}$) values (dimensionless) using the following Eq. (1) described by Jaycock and Parfitt^30^.

1 $${R}_{S}= {A}_{B}\rho \left(\frac{D}{6}\right)$$

where ${A}_{B}$ is the BET surface area measurement, ρ is the solid density, and D is the average particle diameter. Additionally, the density was measured using an automated Micromeritics AccuPyc II 1340 gas pycnometer. The same grinding protocol was used for the single minerals and the model ore to prepare the flotation feed. After grinding and sieving, the samples were thoroughly washed with dilute HCl solution (2%) to clean the particle surfaces.

### Single mineral flotation

Single mineral flotation experiments for pure magnetite and quartz were performed using a mini flotation cell (Clausthal cell). In each flotation, 7.5 g of the sample (−106 + 38 µm) was added to the 150 cm^3^ capacity with deionized water. Before the test was performed, the slurry was conditioned with a predetermined amount of PGA for 10 min. Subsequently, reagents (depressant and collector) were added to the suspension and conditioned for 10 (5 + 5) min. Caustic starch was used as a depressant. A fresh 1% alkaline starch solution (1:4 ratio) was prepared for each set of experiments. Lilaflot 822M, recommended and supplied by Nouryon (Sweden), was used as a cationic collector. The pH was adjusted by adding 1.0 M NaOH or 1.0 M HCl. The flotation was carried out for 2 min, scraping every 10 s. The froth products and tails were collected, weighed, dried, and recovery was calculated based on the dry weight. Each experiment was performed in duplicate and the average was reported.

### Mixed mineral flotation

The mixed mineral flotation on the model ore was carried out using the same cell. The model ore consisted of 5.0 g magnetite and 2.5 g quartz (ratio 2:1). 7.5 g of the mixture (−106 + 38 µm) was used with deionized water was used for each test. The same procedure, such as the single mineral flotation, was also considered for the reverse flotation. The collector was fixed at 300 g/t, and the depressant was varied together with GAs. Conditioning was performed for 10 min followed by flotation for 2 min. The froth products and tails were collected, weighed, dried, and recovery was calculated based on the dry weight and chemical analyses using induction plasma (ICP OES). Each experiment was performed in duplicate and the average was reported. The separation efficiency (S.E) for each test was calculated using Eq. (2)^31^. Where *f*, *c*, and *t* are the feed, concentrate, and tail grades of iron, respectively, a higher S.E value extrapolates a better separation efficiency of the process.

2 $$\mathrm{S}.\mathrm{E }\left(\mathrm{\%}\right)= \frac{\mathrm{c}(\mathrm{f}-\mathrm{t})(\mathrm{c}-\mathrm{f})(100-\mathrm{t})}{\mathrm{f}{\left(\mathrm{c}-\mathrm{t}\right)}^{2}(100-\mathrm{f})}\times 100$$

### Zeta potential measurements

Zeta potentials of the samples were measured using a CAD ZetaCompact instrument. 20 mg of finely ground samples (−5 µm) was mixed with 50 ml of deionized water together with predetermined reagents in a beaker. The background electrolyte was a 10^–2^ M KCl solution. The pH was adjusted by using an HCl or NaOH solution. The mixture was stirred with a magnetic stirrer for 10 min and left to stand. The suspension supernatant was then transferred to an electrophoresis cell using a syringe. The particles in the suspension were illuminated by a laser and their electrophoresis was observed by a camera. Video analysis is done with Zeta4 software based on the Smoluchowski Eqiuation^32,33^ to calculate the zeta potential from electrophoretic mobility data^34^. The reported result for each data point is an average of three measurements with different aliquots.

### Adsorption measurements

**Figure 3:**
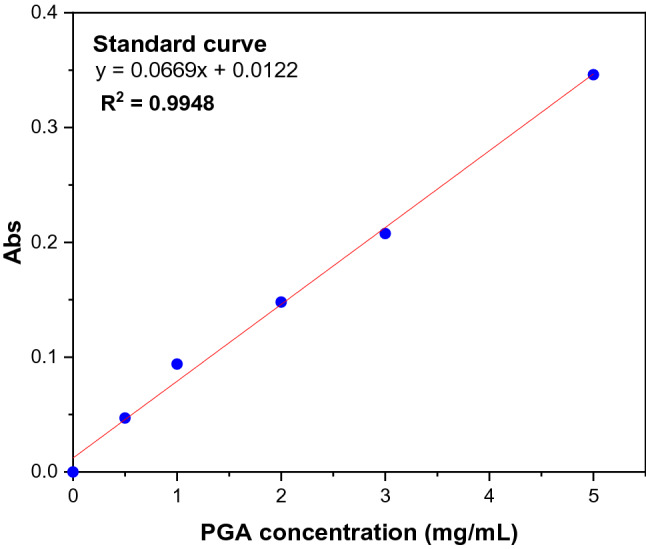
Standard curve of PGA adsorption.

Adsorption measurements to determine the amount of adsorbed PGA were carried out using the solution depletion method on the UV–VIS spectrometer (DU Series 730 – Beckman Coulter, USA). Standard solutions with PGA concentrations ranging from 0.5 to 5 mg/ml were used to obtain the calibration curve (Fig. 3). For the measurements, the maximum absorbance at 220 nm was used. 1.0 g of the sample (−106 + 38 µm) with 40 ml and the predetermined reagent concentration were added to a 100 ml flask. The suspension was stirred for 2 h at pH 10 and 20 ± 1 °C to ensure maximum adsorption. After vacuum filtration, the solution was passed through a 0.22 µm millipore membrane. The concentration of the remaining PGA in the solution was analyzed using UV absorbance at a wavelength of 220 nm. The measurements were corrected for the blanks and performed in triplicate. The concentration that was depleted from the solution was assumed to be adsorbed onto the surface of the sample particle. The adsorption density was calculated using Eq. (3);

3 $${Q}_{e}=\frac{\left({C}_{1}-{C}_{0}\right)V}{m}$$

where ${Q}_{e}$ is the amount of PGA (mg/g) adsorbed on the sample particle surface, ${C}_{0}$ and ${C}_{1}$ are the initial and final concentrations, i.e., before and after adsorption (mg/L), respectively. *m* is the mass (g) of the sample, and *V* is the volume (L) of the PGA solution. Furthermore, the experimental data for the adsorption isotherms were fitted to the Langmuir (Eq. 4) and Freundlich (Eq. 5) models:

4 $${Q}_{e}=\frac{{K}_{L}{C}_{e}{Q}_{0}}{1+{K}_{L}{C}_{e}}$$

5 $${Q}_{e}={K}_{F}{C}_{e}^\frac{1}{n}$$

where ${Q}_{e}$ is the amount of PGA (mg/g) adsorbed, ${C}_{e}$ is the equilibrium concentration of PGA. ${Q}_{m}$ and ${K}_{L}$ are Langmuir constants whilst ${K}_{F}$ and 1/*n* are the Freundlich constants related to maximum monolayer adsorption capacity and energy of adsorption, respectively^35^.

### Stability measurements

Stability measurements of suspensions were performed using Turbiscan LAB EXPERT (Formulaction, France). Measurements were carried out to determine the behavior of coagulation and dispersion in the presence and absence of PGA. 50 mg of quartz and magnetite were added separately to 40 mL of deionized water. A predetermined amount of reagents was then added and stirred for 20 min at pH 10. 20 mL of suspension was transferred to a measuring vial and scanned at the height of 40 mm at 30° C. The suspension was scanned over 100 times in 60 min at 30 s intervals. The intensities of transmission (T) and backscattering (BS) of pulsed near-infrared light (λ = 880 nm) were recorded as a function of time. The data was then analyzed using TLab EXPERT 1.13 and Turbiscan Easy Soft software to calculate the Turbiscan Stability Index (TSI) Eq. (6). Where ${x}_{i}$ is the average backscattering for a minute of measurement, ${x}_{BS}$ is the average ${x}_{i}$, and $n$ is the number of scans. The TSI coefficient values vary from 0 to 100, translating into an extremely stable to an unstable system^36,37^.

6 $$TSI= \sqrt{\frac{\sum_{i=1}^{n}{({x}_{i}-{X}_{BS})}^{2}}{n-1}}$$

### FT-IR spectroscopy measurements

PGA characterization was performed using Fourier transform infrared (FTIR) spectroscopy with attenuated total reflection (ATR) attachment. The samples were ground to approximately −2 µm using an agate mortar and pestle. A 2.0 g mineral sample was treated with predetermined reagents and conditioned for 40 min at pH 10. The solid samples were thoroughly washed using deionized water. After washing and vacuum drying at 35 °C for 24 h, the samples were subjected to FTIR analysis. The samples were analyzed by diffuse reflectance (DR) and ATR-FTIR spectroscopy, using an IFS 66 V/S instrument and a Vertex 80v instrument, respectively (Bruker Optics, Ettlingen, Germany) under vacuum conditions (below 7 mbar), according to the protocol by András and Björn^38^. For diffuse reflectance measurements, powder from dry samples (ca. 10 mg) was mixed with infrared spectroscopy grade potassium bromide (KBr, Merck/Sigma-Aldrich, ca. 390 mg) and manually ground using an agate mortar and pestle until a homogeneous mixture was achieved. Spectra were recorded in the range of 400–4000 cm^−1^ at 4 cm^−1^ spectral resolution, and 128 scans were co-added, using pure KBr as the background under the same parameters. Spectra were processed using the built-in functions of OPUS (version 7, Bruker Optics, Ettlingen, Germany). Spectra were the first baseline corrected (64-point rubberband) over the entire spectral range, then vector normalized, and finally offset corrected. After these steps, no smoothing, derivatization, or other processing was applied. ATR measurements were done using a Bruker Platinum accessory with a diamond internal reflection element. Spectra were recorded in the range of 400–4000 cm^−1^ at 4 cm^−1^ spectral resolution, and 100 scans were recorded, using the empty diamond crystal as the background under the same parameters. The spectra were processed using the built-in functions of OPUS (version 7, Bruker Optics, Ettlingen, Germany) in the same way as the DR spectra.

## Results and discussion

### Grinding

**Figure 4:**
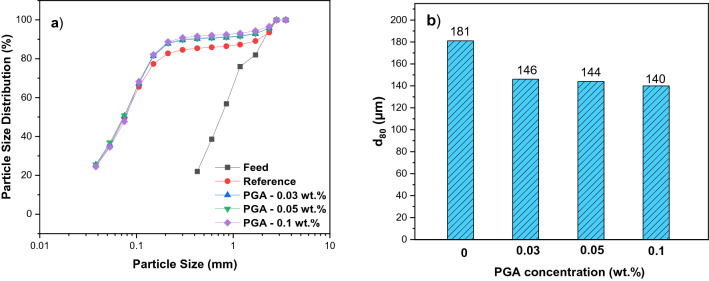
The particle size distribution and d80 of grinding products with and without PGA.

Exploring the d_80_ of ground samples with and without PGA indicated that PGA (in various doses) could provide finer particles (Fig. 4). Further, the introduction of PGA (at 0.1 wt.%) reduced the particle size to a d_80_ = 140 µm compared to 181 µm for the reference, which translates to a finer product. Further size analyses of ground products (Table 2) revealed that the use of PGA could narrow their PSD, for example, the use of PGA (0.1 wt.%) resulted in 56.3% of particles being in + 38–106 µm, while without PGA, 51.2% of the particles were in this size range. This size range mostly favors flotation separation. In other words, the use of PGA could improve the grinding performance expressed by decreasing the distribution of ultrafine (−38 µm) and coarse particles (+ 106 µm) Table 2. Analysis of ground products showed that PGA (0.1%) generated a significantly higher specific surface area (0.89 m^2^/g) than the reference (0.75 m^2^/g). The high specific surface area also translated into a higher surface roughness (${R}_{s}$) of 47.85 for PGA compared to 40.32 for the reference. Similar evidence of the increased surface area and surface roughness with the addition of GAs has been reported by Chipakwe et al.^10^. Evidently, PGA reduced the energy consumption with increasing concentration with a maximum reduction of 31.1% at 0.1 wt.% compared to the reference Table 2. In general, the grinding performance assessment indicated that PGA improved grinding efficiency compared to the reference (without PGA) based on the generation of new surfaces (higher specific surface area), lower energy consumption, and narrowing of the particle size distribution.

**Table 2:** Summary of the grinding test with and without PGA (Ec expressed as work index).

Size range (µm)	Reference	PGA (wt.%)		
	–	0.03	0.05	0.1
+ 106	23.9	21.1	20.0	20.5
+ 38 −106	51.2	54.2	54.7	56.3
−38	24.9	24.7	25.3	23.2
Energy consumption, Ec, (kWh/t)	18.0	14.4	14.3	12.4

### Flotation

#### Single mineral

**Figure 5:**
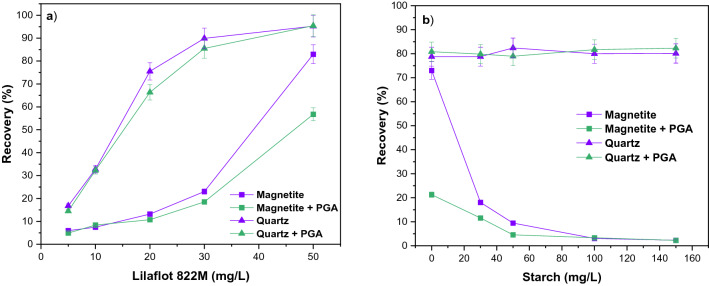
Single mineral flotation of magnetite and quartz as a function of (**a**) collector concentration in the presence and absence of 100 mg/L PGA at pH 10 and (**b**) depressant concentration in the presence and absence of 100 mg/L PGA at pH 10 and collector concentration of 50 mg/L.

Single mineral flotation experiments were carried out to assess the effect of Lilaflot 822M (collector) and starch (depressant) in the absence and presence of PGA (fixed dosage at 100 mg/L). Figure 5a presents the single mineral flotation performance for magnetite and quartz as a function of the collector. With an increase in Lilaflot 822M concentration, the recoveries of magnetite and quartz increased. As expected, the floatability of quartz for both conditions with and without PGA was markedly enhanced by increasing collector dosages (Lilaflot 822M is a silicate collector). In general, the floatability of quartz is comparable, although the presence of PGA resulted in lower floatability at lower collector concentrations. However, in high Lilaflot 822M concentrations, PGA indicated a significant effect on decreasing the floatability of magnetite compared to that of quartz. In other words, these findings suggested that PGA has a depressive effect on magnetite, which may be beneficial considering that magnetite depression is the key in reverse flotation separation. To further explore the impacts of PGA through additional experiments, the concentration of Lilaflot 822M (collector) was fixed at 50 mg/L, where quartz showed its highest floatability (recovery).

Furthermore, the floatability of magnetite and quartz as a depressant function with fixed collector and PGA doses was investigated (Fig. 5b). As anticipated, overly starch and PGA do not affect the floatability of quartz. The floatability of magnetite decreased significantly with increasing starch concentration, confirming the effectiveness of starch as a depressant. For the reference test, the floatability of magnetite continued to decrease with starch addition to a minimum of 2.3% at 100 mg/L. The depressing impact of PGA and reducing its floatability could be detected (Fig. 5b). A significant decrease in magnetite recovery to 21.3% without starch compared to 73.0% for the reference test could confirm the depressing effect of PGA. It can also be seen that the maximum depression effect of starch was observed at 100 mg /L, whilst with the addition of PGA, a comparable depression effect was achieved at 50 mg/L. Generally, single-mineral flotation tests indicated that increasing PGA has a favorable outcome in magnetite depression without changing quartz floatability.

#### Mixed mineral flotation

**Figure 6:**
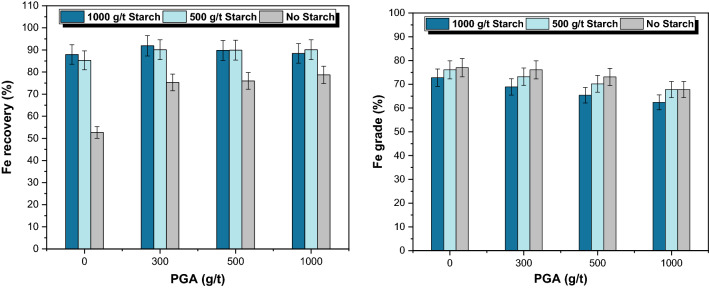
Effect of PGA and starch on magnetite flotation at a fixed amount of collector (300 g/t) at pH 10.

Subsequently, mixed mineral flotation experiments were carried out to assess the effect of PGA on the model ore (magnetite: quartz 2:1 mass ratio). Based on the results of the single mineral flotation, 30 mg/L (which translates to 300 g/t) was considered for the doses of collector, while the starch and PGA dosages varied at pH 10. The variation in magnetite metallurgical recovery (as Fe) as a function of PGA and starch dosage was presented in Fig. 6. The results indicated that Fe recovery (magnetite hydrophilicity) improved by increasing PGA dosage in the absence of starch. However, the improvement was negligible after 300 g/t PGA. In the presence of starch, the recovery variations were insignificant. In other words, no obvious changes could be realized by increasing starch concentration from 500 to 1000 g/t. It could be translated as PGA improving grinding performance and reducing depressant consumption.

Higher Fe grades were reported in the absence of starch, although the recoveries are generally lower (Fig. 6). Therefore, the separation efficiency (SE) was calculated to better understand the interaction of PGA and starch and their resulting synergistic effects on separation (Table 3). Flotation outcomes indicated that PGA (in all dosages) could enhance the S.E in the absence of starch. 300 g/t PGA (in the absence of starch) provided results similar to those of 1000 g/t starch (in the absence of PGA). In general, the presence of both PGA and starch could improve the S.E compared to the reference conditions. It can be observed that high doses of both PGA and starch were not desirable. The highest S.E can be observed when starch and PGA were 50 and 300 g/t, respectively. The observed improvements in flotation separation corroborate findings reported elsewhere on the beneficial effects of a narrow particle size distribution^27,28^ and surface roughness^39,40^. Besides the superior properties observed from using PGA, surface analyses were considered to assess PGA interaction with mineral surfaces.

**Table 3:** Variation of Separation Efficiency with and Without PGA and Starch.

Separation efficiency (%)			
PGA (g/t)	No starch	500 (g/t) starch	1000 (g/t) starch
0	43.7	58.0	56.8
300	56.1	60.6	50.8
500	53.1	52.9	43.7
1000	47.0	48.5	37.6

### Zeta potential measurements

**Figure 7:**
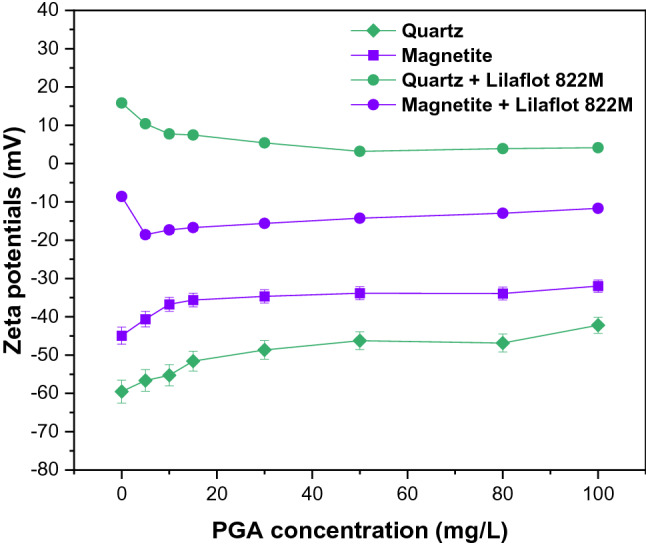
Zeta potentials at varying PGA concentration with fixed Lilaflot 822M (50 mg/L) and pH.

Zeta potential measurements were carried out to further explore the interaction mechanism between PGA, Lilaflot 822M, and mineral particles to understand the observed flotation behavior. It is important to assess how these surfactants change the surface properties that affect the flotation behavior. Zeta potential measurement results indicated (Fig. 7) that the addition of PGA slightly affects the electrical charge on the surface of both quartz and magnetite implying a change in either solution or surface chemistry or both. These negligible effects could be due to the nonionic PGA composition. The zeta potentials for quartz decreased (absolute value) after PGA treatment. The evaluations illustrated that the zeta potentials decreased rapidly from 0 to 15 mg/L (PGA concentration) for both minerals, with quartz changing from −59.5 to −51.6 mV (Δζ ~  + 7.9 mV) while magnetite changed from −44.9 to −35.6 mV (Δζ ~  + 9.3 mV). The ζ measurements demonstrated that the addition of PGA to both minerals above 30 mg/L has almost no further effect in the investigated ranges. The addition of Lilaflot 822M to the treated minerals results in a behavior change to give more positive zeta potentials, especially for quartz. This illustrated that PGA had an insignificant effect on quartz, evident from the marked effect of Lilaflot 822M adsorption on the surface as a collector. A similar behavior could be observed with the addition of PGA, where the zeta potentials decreased with increasing PGA concentration. The relatively smaller change in magnetite zeta potentials, compared to quartz after Lilaflot 822M treatment, indicated that the presence of PGA reduced the interaction between Lilaflot 822M and magnetite. This highlighted that PGA adsorbed on magnetite rather than the quartz surface based on the collector impact.

### Stability measurements

**Figure 8:**
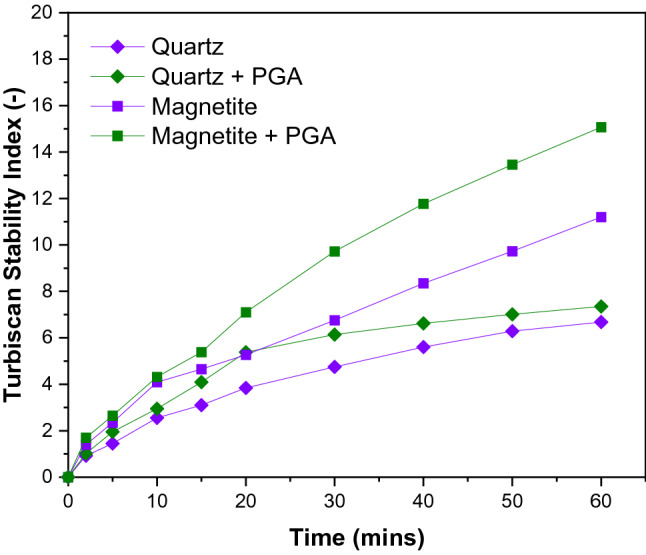
Stability of magnetite and quartz suspensions in the absence and presence of PGA (100 mg/L and pH 10).

The Turbiscan stability index (TSI) assessments (Fig. 8) showed that treatment of both particle surfaces results in decreased stability compared to the reference (without PGA treatment). The observations are expected for any suspension, as the destabilization illustrated the effect of flocculation, coagulation, sedimentation, coalescence, and even a combination^36,37^. Figure 8 showed the destabilization kinetics of magnetite and quartz suspensions as a function of time. TSI values demonstrated that a relatively stable system was in agreement with the zeta potential results (Fig. 7) for both quartz and magnetite, which are all below −30 mV at pH 10, showing high stability^41,42^. The TSI values for magnetite are higher compared to those of quartz, generally showing less stability. After PGA treatment, the stability variation was more pronounced for magnetite compared to quartz for the total investigated time of 60 min. In other words, these outcomes suggested that the destabilizing effect of PGA was more pronounced on magnetite than on quartz, pointing to increased adsorption. This is consistent with the zeta potentials, which showed a higher absolute value for quartz relative to magnetite, indicating better suspension stability. When examining magnetite particles, the increase in TSI values (reduced dispersion) could help explain the depression effect of PGA, which might be due to aggregation/flocculation, thus hindering flotation. Similar observations have been reported in which polysaccharides interact with iron oxides from aggregations^43,44^.

### Adsorption test

**Figure 9:**
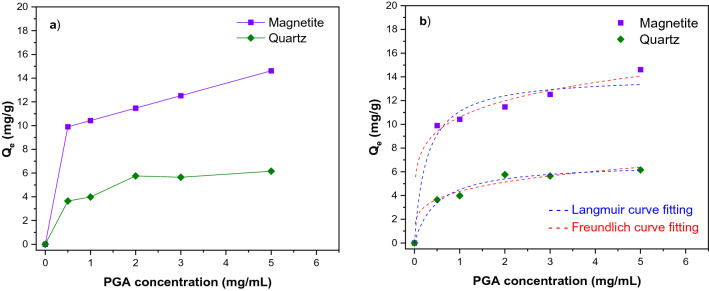
Adsorption of PGA as a function of initial concentration at pH 10.

Figure 9a showed an increase in adsorbed PGA per unit mass of magnetite and quartz. Furthermore, it could be observed that the adsorption capacity of magnetite was more than double that of quartz. For magnetite, a trend of continued increase can be demonstrated based on the still high slope beyond 5 mg/ml, while for quartz, the curve started plateauing after 2 mg/ml. The adsorption isotherms of PGA in magnetite and quartz particles are shown in Fig. 9b. The results of the adsorption isotherms using the depletion method were fitted to the Langmuir (Eq. 2) and Freundlich (Eq. 3) models and are summarized in Table 4. The Langmuir model gave the best fit with R^2^ of 0.9651 and 0.9721, whilst the Freundlich model had R^2^ of 0.9085 and 0.8506 for magnetite and quartz, respectively. The trends observed in Fig. 9a were also supported by the calculated parameters from the Langmuir and Freundlich models (Table 4). The obtained parameters $n$ and ${Q}_{m}$ values (highlighted the strength and capacity of the adsorption, respectively) were higher for magnetite compared to quartz, which suggested that the adsorption of PGA on magnetite was much stronger. The findings from the adsorption studies showed that PGA fairly adsorbs on both magnetite and quartz, further confirming the effect of PGA on single-mineral flotation, possibly reducing the surface areas available for collector adsorption, especially for magnetite. These outcomes corroborated the zeta potentials and stability measurement findings that magnetite had a higher and stronger adsorption capability for PGA compared to that of quartz.

**Table 4:** Langmuir and Freundlich parameters for PGA adsorption on magnetite and quartz.

Particles	Langmuir equation			Freundlich equation		
	${Q}_{m}$	${K}_{L}$	${R}^{2}$	$n$	${K}_{F}$	${R}^{2}$
Magnetite	12.10	$9\times {10}^{-1}$	0.9651	6.00	3.43	0.9085
Quartz	5.85	$6.6\times {10}^{-3}$	0.9721	5.18	1.25	0.8506

### FTIR spectra analysis

**Figure 10:**
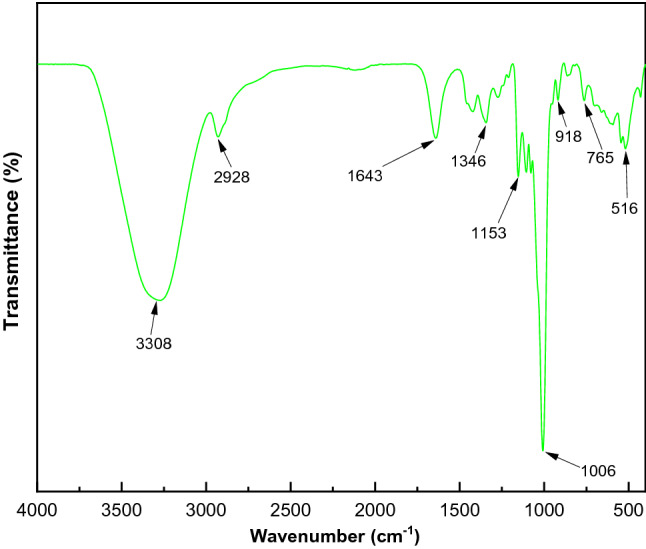
FT-IR spectra of the examined PGA using ATIR.

FTIR was used to characterize the functional groups in PGA, which is mainly a dextran (Fig. 10). The main characteristic peaks showed a broad peak between 3000 and 3600 cm^−1^, demonstrating a hydroxyl group^45,46^—OH stretching vibration and appearing at 3308 cm^−1^. A distinct characteristic peak appeared at 2928 cm^−1^, which was related to the C-H stretch vibration in the sugar ring^45,47^. Furthermore, the C–O stretching vibration was illustrated at 1643 cm^−1^^48^. A peak emerged at 1346 cm^−1^ that could be assigned to the symmetric CH_3_ bending^46^. Strong characteristic peaks emerged at 1006 cm^−1^ and 918 cm^−1^ in the region 950–1100 cm^−1^_,_ which was attributed to the C–O–C and C–O groups of polysaccharides^46^. The peak in the region 950–1100 cm^−1^ was due to the presence of the (1 → 6)- and (1 → 3)-linked α-D-glucose units, respectively^49,50^.

**Figure 11:**
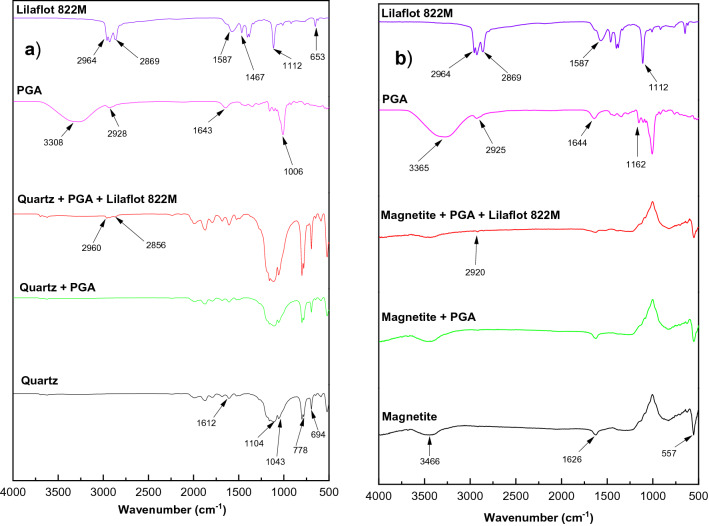
FTIR spectra of (**a**) quartz and (**b**) magnetite in the presence and absence of PGA and Lilaflot 822M (at 100 mg/L).

The adsorption mechanism of PGA was investigated together with the collector on both magnetite and quartz surfaces. Figure 11a showed the spectra for pure quartz, quartz + PGA, quartz + PGA + Lilaflot 822M together with the respective pure reagents. For Lilaflot 822M, characteristic peaks emerged at 2964 cm^−1^, 2869 cm^−1^, which were attributed to the CH_2_ stretching bond of the acyclic compounds^51^. The peak at 1587, 1464 and 653 cm^−1^ could be attributed to the bending of the NH_2_ or NH bonds^51–53^. It is evident from Fig. 11a that the presence or absence of PGA on the quartz surface had no effect, as no observable change in the spectra exists. After treatment with Lilaflot 822M, a characteristic peak was observed on the quartz surface. After treatment of quartz with Lilaflot 822M, the characteristic peak of OH at 2964 cm^−1^ shifted to 2960 cm^−1^ as observed in quartz + PGA + Lilaflot 822M, which was consistent with the findings reported by Liu et al.^51^. Furthermore, the characteristic stretching of CH at 2869 cm^−1^ also changed to 2856 cm^−1^ after treatment. This indicated that Lilaflot 822M adsorbed onto the quartz surface through the OH and CH bonds. Compared to Lilaflot 822M, PGA did not show a characteristic peak and, given the water washing in the procedure, which meant that Lilaflot 822M adsorbed chemically and collaborated with the finding documented by Huang et al.^52^ and Liu et al.^51^ on the adsorption of amines on the quartz surface. In contrast, the absence of a characteristic PGA peak on the quartz surface suggested that PGA did not chemically adsorb on the quartz surface. This points to the slightly weak physisorption of PGA on the quartz surface.

The FTIR spectra for magnetite in the presence and absence of PGA and Lilaflot 822M (Fig. 11b) indicated that there was no characteristic peak in magnetite treated with PGA, and therefore there was no impact from PGA. Moreover, Lilaflot 822M had a negligible impact on the magnetite surface, evident from the relatively weak characteristic shifted peak at 2920 cm^−1.^ Negligible characteristic peaks on magnetite meant weak adsorption, especially compared to Lilaflot 822M adsorption on the quartz surface. The absence of a characteristic peak on magnetite in the presence of PGA suggested that PGA did not adsorb chemically but rather possibly a physical interaction. From the IR analysis, it can be said that for both quartz and magnetite PGAs, the interaction mechanism was not chemical. However, considering that the particles were subjected to thorough washing with water before analysis, the interaction could be physical and probably due to hydrogen bonding. Shrimali and Miller^22^, in their concise review of the interaction of polysaccharides and iron ores, outline that polysaccharide adsorption may be due to hydrogen bonding, hydrophobic interaction, or chemical complexing (acid–base reaction). The hydrophobic interaction might play a major role in reducing the surface charge, allowing for flocculation/aggregation of the particles; thus, leading to magnetite depression. This is consistent with observations suggested that the mechanism of nonionic polymer adsorption would be due to the hydrophobic chain interaction leading to the bridging and/or charge neutralization^23,54,55^.

## Conclusions

In this study, a novel polysaccharide-based grinding aid was first used to improve grinding performance, and its secondary effects on reverse flotation of magnetite quartz from magnetite were explored. Empirical observations on batch dry grinding indicate that PGA improved grinding efficiency by reducing energy consumption, a narrower PSD, increased specific surface area, rougher surfaces, and a finer PSD. According to the single mineral flotation tests, PGA has a depressing effect (positive effects) on magnetite particles with a negligible effect on quartz particles. Through mixed mineral flotation separation (magnetite + quartz at 2:1) comparable results of 86% recovery and Fe grade of 62% could be achieved using PGA only without starch. For the best balance in recovery, grade and separation efficiency, 500 g/t starch could be recommended along with 300 g/t PGA. Based on UV–vis spectra, zeta potential tests, Fourier transform infrared spectroscopy (FT-IR), and stability measurements, the adsorption mechanism is mainly via physical interaction. In other words, the improved flotation separation efficiency in the presence of PGA could also be attributed to the narrowing of the particle size distribution and the increase in surface roughness. These results highlighted that the selection of suitable grinding aids (ecofriendly) could potentially reduce energy consumption (decrease CO_2_ emissions), improve the distribution of suitable particles for downstream processes, and has no negative chemical impacts (even positive effects) on the separation stages.
