# Author Correction: Beneficial effects of a polysaccharide-based grinding aid on magnetite flotation: a green approach

**DOI:** 10.1038/s41598-023-32197-0

**Published:** 2023-04-05

**Authors:** Vitalis Chipakwe, Tommy Karlkvist, Jan Rosenkranz, Saeed Chehreh Chelgani

**Affiliations:** Minerals and Metallurgical Engineering, Department of Civil, Environmental and Natural Resources Engineering, Luleå University of Technology, 971 87 Luleå, Sweden

Correction to: *Scientific Reports* 10.1038/s41598-022-10304-x, published online 20 April 2022

The Acknowledgements section in the original version of this Article was incomplete.

“This manuscript resulted from a project financially supported by CAMM, the Center of Advanced Mining and Metallurgy, as a center of excellence at the Luleå University of Technology. The authors also thank Solenis for their help with the reagents used as grinding aids in this study. The authors express their gratitude to Dr. Andras Gorzsas from Umeå University for assistance with FTIR analysis and fruitful discussions.”

now reads:

"This manuscript resulted from a project financially supported by CAMM^2^, the Center of Advanced Mining and Metallurgy, as a center of excellence at the Luleå University of Technology, and Vinnova for the RIO-MUN (Reverse flotation of iron oxide using magnetic, ultrasonic, nanobubble technology), project number: 2020-04835. The authors also thank Nouryon (Sweden) and Solenis for their help with the reagents used in this study. The authors express their gratitude to Dr. Andras Gorzsas from Umeå University for assistance with FTIR analysis and fruitful discussions."

The original Article has been corrected.

